# A Modified Sagittal Spine Postural Classification and Its Relationship to Deformities and Spinal Mobility in a Chinese Osteoporotic Population

**DOI:** 10.1371/journal.pone.0038560

**Published:** 2012-06-05

**Authors:** Hua-Jun Wang, Hugo Giambini, Wen-Jun Zhang, Gan-Hu Ye, Chunfeng Zhao, Kai-Nan An, Yi-Kai Li, Wen-Rui Lan, Jian-You Li, Xue-Sheng Jiang, Qiu-Lan Zou, Xiao-Ying Zhang, Chao Chen

**Affiliations:** 1 Department of Orthopedics, School of Traditional Chinese Medicine, Southern Medical University, Guangzhou, China; 2 Biomechanics Laboratory, Division of Orthopedic Research, Mayo Clinic, Rochester, Minnesota, United States of America; 3 Chang Ping Hospital, Dongguan, China; 4 Orthopedic Department, Huzhou Central Hospital, Huzhou, China; 5 You-Hao Residential Care Home, Guangzhou, China; Faculté de médecine de Nantes, France

## Abstract

**Background:**

Abnormal posture and spinal mobility have been demonstrated to cause functional impairment in the quality of life, especially in the postmenopausal osteoporotic population. Most of the literature studies focus on either thoracic kyphosis or lumbar lordosis, but not on the change of the entire spinal alignment. Very few articles reported the spinal alignment of Chinese people. The purpose of this study was threefold: to classify the spinal curvature based on the classification system defined by Satoh consisting of the entire spine alignment; to identify the change of trunk mobility; and to relate spinal curvature to balance disorder in a Chinese population.

**Methodology/Principal Findings:**

450 osteoporotic volunteers were recruited for this study. Spinal range of motion and global curvature were evaluated noninvasively using the Spinal-Mouse® system and sagittal postural deformities were characterized.

**Results:**

We found a new spine postural alignment consisting of an increased thoracic kyphosis and decreased lumbar lordosis which we classified as our modified round back. We did not find any of Satoh’s type 5 classification in our population. Type 2 sagittal alignment was the most common spinal deformity (38.44%). In standing, thoracic kyphosis angles in types 2 (58.34°) and 3 (58.03°) were the largest and lumbar lordosis angles in types 4 (13.95°) and 5 (−8.61°) were the smallest. The range of flexion (ROF) and range of flexion-extension (ROFE) of types 2 and 3 were usually greater than types 4 and 5, with type 1 being the largest.

**Conclusions/Significance:**

The present study classified and compared for the first time the mobility, curvature and balance in a Chinese population based on the entire spine alignment and found types 4 and 5 to present the worst balance and mobility. This study included a new spine postural alignment classification that should be considered in future population studies.

## Introduction

Osteoporosis, leading to an increased risk of fracture, poor posture and reduced functional ability is a significant global public health issue which has affected more than 200 million people and is expected to substantially increase by the year 2050 [1]. In the year 2005, approximately $19 billion was spent in osteoporosis related fractures, and by the year 2025, the cost is estimated to reach $25.3 billion (National Osteoporosis Foundation). The most common clinical manifestation of osteoporotic fractures are vertebral fractures. Older female patients are more severely affected due to the compromised resistance of bone as a consequence of decreased bone mineral, reduced bone quality and destructive micro architecture resulting from post-menopause [2], [3].

In addition to the above bone characteristic, more attention has been drawn into studies involving functional impairment including curvature deformity, balance disorder and the change of trunk mobility [3]–[19]. Such abnormal posture and spinal mobility is demonstrated to cause significant functional impairments in activities of daily living [3], [11], [15]. A series of studies by Miyakoshi et al. suggested lumbar kyphosis as a negative predictor of quality of life (QOL) and spinal mobility as a positive predictor and the most important factor relating QOL [15]. In addition, lumbar spinal mobility was proven to be the most important factor to QOL in patients with postmenopausal osteoporosis [13]. Conversely for middle-aged and elderly males, sagittal balance, lumbar lordosis angle, and spinal range of motion were also proved to be related to QOL [6]. On the other hand, studies have shown that thoracic hyperkyphosis is independently associated with decreased mobility and accompanied by a slower gait, poor balance, and greater body sway, which in turn is correlated with an increased tendency to falls [9], [10], [17]. Moreover it was reported that trunk deformities and spinal mobility also induce chronic back pain, increase vertebral fractures risk, reduce gait and stair-climbing function due to a decrease in lung function, and increase mortality rates, decreasing QOL and life satisfaction [5], [7], [16], [19]. Therefore, rehabilitation intervention which has showed to influence a reduction in kyphosis may be an effective way to improve daily living functionality and QOL [4], [18].

However an explanation to abnormal posture, spinal mobility and balance is multiplex and multifactorial. The proportion of older persons with the worst degrees of kyphosis who have vertebral fractures is only 36–37% [20]. Other causes impacting hyperkyphosis include postural changes, muscular weakness, degenerative disc disease and some genetic predisposition [20]–[23]. Consequently, there still exist some controversies which are not yet fully understood. Although lumbar lordosis tends to decrease with age in most research studies [22], [24] other reports are inconsistent, reporting an increase [24] or no change in curvature [25], whereas Takahashi et al. showed that 11.9% of the participants had a decreased lumbar lordosis, and 4.7% exhibited an increased lumbar curvature [19]. While studies have demonstrated thoracic hyperkyphosis as an independent predictor of balance and QOL [6], [9], [17], lumbar kyphosis has been shown to affect spinal inclination and postural balance, presenting an additional risk factor for a tendency to falls [8], [13], [26]. Most notably, abnormal posture and spinal mobility should be studied as an overall alignment pattern including the thoracic and lumbar regions of the spine [15], [27], [28]. A same angular change in a similar segment of different persons may have a different effect on the global spine due to the compensatory and interactive relationship among separate segments of the spine in the process of senescence. Thus, it is important and meaningful to focus more attention on changes and relationships between different global spine curvature types [15], [27], [28]. Meanwhile a difference has been reported in the shape of the sagittal spinal curvature between Japan and the United States [29]. With one of the biggest populations in the world, a large elderly population and increasing longevity, osteoporosis has become a significant burden on society and healthcare systems in China [30]. An understanding of the changes of spinal deformity and functional impairment in the Chinese population would be useful in the planning of public health strategies in this region. However, there are very few articles reporting spinal functional impairment and alignment in Chinese people.

Thus, the objective of this study was to provide further evidence about the change of trunk mobility and the relationship between spinal curvature and balance disorder, especially for the different type of global spine deformity in a Chinese population.

## Materials and Methods

### Ethics Statement

Informed consent was obtained from all participants prior to examination, and ethical approval to undertake this study was obtained from Human Research Ethics Committee, Southern Medical University.

### Participants

For this cross-sectional study, a total of 476 elderly women volunteers, over 60 years of age, with osteoporosis were recruited from local community centers. Diagnosis of osteoporosis was made according to the World Health Organization criteria defined by a bone mineral density (BMD) T-score of at least 2.5 standard deviations below the young normal sex-matched BMD of the reference database**.** In addition, participants were questioned about their medical history and were excluded if they had a history of neurologic and musculoskeletal disease such as acute or severe chronic back pain within the last 6 months that required medical attention or treatment, documented vertebral fractures within the last 6 months, previous surgery of the spine, dislocations of the spine, spondylolisthesis, spondylolysis, hip fractures, metastases, and rheumatologic disorder. Participants with any other possible disorder affecting bone metabolism were also excluded. Finally, 450 volunteers (mean 75 yrs., range 60–95) were eligible and joined our study.

### Spine Range of Motion and Global Curvature Measurements

Using the Spinal-Mouse® we were able to evaluate spine range of motion (ROM) and global curvature (Idiag, Volkerswill, Switzerland). This is an electronic computer-aided device that measures sagittal spinal ROM and intersegmental angles noninvasively using a surface technique. The intra-class coefficients for curvature measurement with Spinal-Mouse® are 0.92–0.95 [31]. To avoid inter-measure variation, all the measurements were done by one examiner who was experienced in assessing spinal function using the Spinal-Mouse® system. Each measurement was conducted three times and the mean value obtained.

Spine curvature, spine inclination (angle of the plumb line bisecting the trochanter major and running through the middle of the supporting area of the feet) and sacral inclination angle (Sac/Hip: sacral slope defined as the angle between the horizontal and the sacral plate) were evaluated in the neutral upright position by sliding of the Spinal-Mouse® along the spine. All spine data were calculated and displayed on the computer automatically. Thoracic kyphosis was expressed as a positive value and lumbar lordosis expressed as a negative value. This process was repeated with the subject in a maximum bending position and a maximum extension position allowing for measurement of spinal mobility. Balance was related to spine inclination and the entire spine alignment measured by the angle of the whole trunk. A large angle indicated worst balance.

### Postural Classification and Comparison

**Figure 1:**
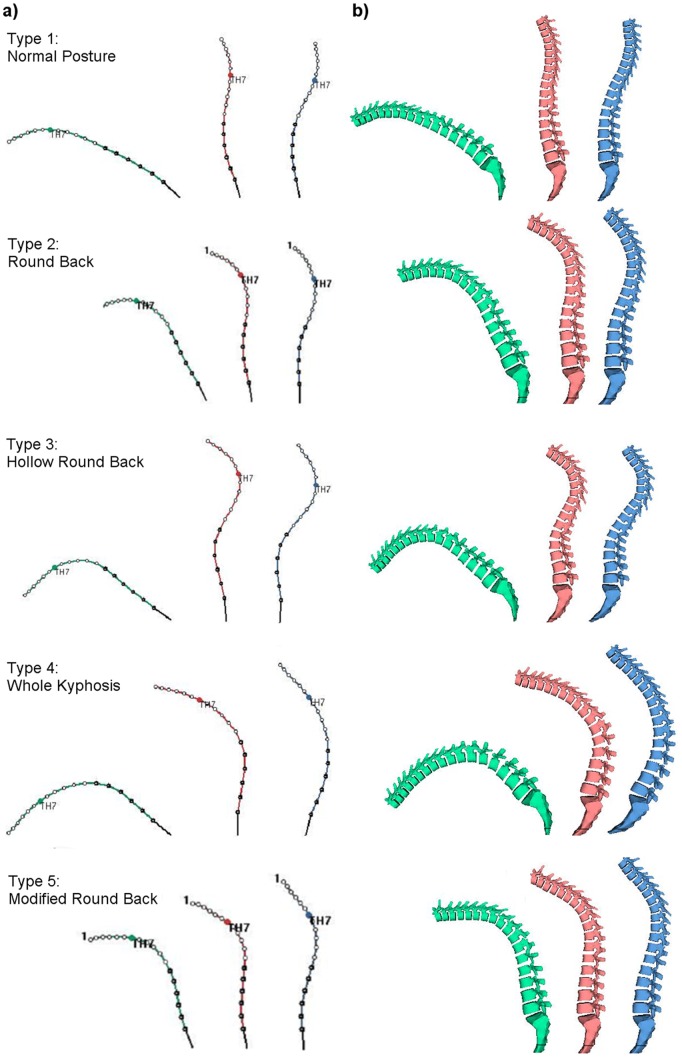
Sagittal spine alignments in flexed, standing and extended positions as acquired using the Spinal-Mouse® System. a) Type 1: Normal Posture; Type 2: Round Back; Type 3: Hollow Round Back; Type 4: Whole Kyphosis; Type 5: Modified Round Back. b) Representation of the different postural types in spine form.

Classification of postures was made based on the visual curvature of the spine of the volunteers, palpation of the spine and curvature results from the spinal mouse®. Sagittal postural deformities were classified by two trained spine surgeons, and upon disagreement, a third spine surgeon was consulted before a final judgment was made. Sagittal postures were divided into the following five groups based on the entire spine alignment according to the classification proposed by *Satoh et al*. [27]: 1) Normal Posture (NP): without apparent change in spinal curve; 2) Round Back (RB): with increased thoracic kyphosis and normal lumbar lordosis; 3) Hollow Round Back (HRB): with increased thoracic kyphosis and lumbar lordosis; 4) Whole Kyphosis (WK): with extensive kyphosis from the thoracic to the lumbar spine and 5) Lower Acute Kyphosis (LAK): with localized lumbar kyphosis and a straight thoracic spine (*not found*) (Figure 1).

### Data Analysis and Statistics

All data are presented as mean and standard deviation (SD) and were analyzed using the Statistical Package for Social Sciences (SPSS, Chicago, IL; version 13.0). Descriptive statistics was used to describe the demographic and measurement variables of all the subjects. Categorical variables were expressed as frequencies and percentages for each variable. Continuous variables were presented as mean values±SD. The factorial design ANOVA and Student Newman Keuls was applied for a comparison between posture types. A *P*-*value* <0.05 was considered statistically significant.

## Results

Volunteers were classified into five types according to Satoh’s classification system. Notably, the type 5 (Lower Acute Kyphosis (LAK): localized lumbar kyphosis and a straight thoracic spine) was not found in our population but rather a new spine alignment was found consisting of an increased thoracic kyphosis and decreased lumbar lordosis which we classified as our modified type 5 and named Modified Round Back (MRB). Among the classified spines, types 2 (38.44%) and 5 (29.33%) sagittal alignment were the most common deformity with type 4 (4.44%) being the least common (Table 1).

**Table 1:** Summary of data for all curves types and conditions.

Table 1. Spinal Curvature and Mobility Comparison						
	Type 1	Type 2	Type 3	Type 4	Type 5	Total
N (%)	50 (11.11%)	173 (38.44%)	75 (16.67%)	20 (4.44%)	132 (29.33%)	450 (100%)
Age	73.34 (6.98)	74.26 (7.80)	71.84 (7.17)	81.45 (7.10) ^a, b, c^	78.36 (7.29) ^a, b, c, d^	75.28 (7.87)
**Thoracic spine**						
Standing	39.24 (4.22)	58.64 (10.40)^a^	58.03 (8.63)^a^	51.55 (14.93)^a, b, c^	52.32 (11.61)^a, b, c^	54.21 (11.86)
ROF	16.90 (10.54)	8.30 (8.76)^a^	8.36 (9.25)^a^	3.65 (8.39)^a, b, c^	9.15 (11.06)^a, d^	9.31 (10.13)
ROE	−5.10 (10.76)	−5.14 (10.03)	−5.84 (7.55)	−8.55 (9.20)	−5.74 (8.24)	−5.58 (9.19)
ROFE	21.90 (14.07)	13.42 (12.90)^a^	14.16 (11.44)^a^	12.15 (10.45)^a^	14.86 (14.16)^a^	14.85 (13.29)
**Lumbar spine**						
Standing	−22.58 (4.82)	−21.49 (3.29)	−31.61 (3.45)^a, b^	13.95 (12.68)^a, b, c^	−8.61 (5.10)^a, b, c, d^	−17.95 (11.51)
ROF	42.54 (9.54)	37.60 (12.91)^a^	43.35 (13.52)^b^	17.80 (13.35)^a, b, c^	26.88 (11.64)^a, b, c, d^	35.08 (14.29)
ROE	−5.62 (5.70)	−5.43 (5.96)	−4.11 (5.46)	−6.40 (6.03)	−5.65 (5.72)	−5.34 (5.79)
ROFE	48.12 (10.16)	43.02 (15.44)	47.36 (15.46)	24.25 (14.08)^a, b, c^	32.41 (13.59)^a, b, c, d^	40.36 (15.88)
**Whole trunk**						
Standing	1.82 (3.21)	6.10 (6.71)^a^	2.99 (5.89) b	22.50 (15.84)^a, b, c^	10.20 (7.38)^a, b, c, d^	7.04 (8.38)
ROF	89.88 (19.17)	75.28 (22.52)^a^	85.92 (21.09)^b^	54.95 (29.35)^a, b, c^	63.58 (25.42)^a, b, c^	74.34 (25.16)
ROE	−18.20 (6.34)	−16.56 (5.82)	−16.63 (5.69)	−14.00 (8.07)	−15.63 (6.53)	−16.37 (6.22)
ROFE	108.00 (21.93)	91.73 (25.19)^a^	102.51 (23.16)^b^	68.95 (32.73)^a, b, c^	79.13 (28.07)^a, b, c, d^	90.63 (27.87)
**Sac/Hip**						
Standing	11.32 (5.20)	8.16 (5.93)^a^	13.96 (6.75)^a, b^	−2.35 (6.89)^a, b, c^	4.08 (6.68)^a, b, c, d^	7.81 (7.44)
ROF	49.96 (17.68)	42.38 (16.77)	47.92 (16.97)	39.60 (22.68)^a^	39.00 (20.46)^a^	43.03 (18.65)
ROE	−11.10 (7.75)	−9.47 (5.52)	−10.48 (5.17)	−6.05 (6.39)^a, b, c^	−8.82 (5.69)^d^	−9.48 (5.91)
ROFE	60.94 (20.99)	51.81 (18.20)	58.35 (17.42)	45.65 (24.18)^a, c^	47.77 (21.75)^a, c^	52.45 (20.25)

Type 1: Normal Posture; Type 2: Round Back; Type 3: Hollow Round Back; Type 4: Whole Kyphosis; Type 5: Modified Round Back.

**Standing**: Angle in standing position; **ROF**: Range of Flexion; **ROE**: Range of Extension; **ROFE**: Range of Flexion and Extension.

**Whole trunk** (Spinal Inclination): angle of the plumb line which bisects the trochanter major and runs through the middle of the supporting area of the feet.

**Sac/Hip**: Sacral slope defined as the angle between the horizontal and the sacral plate.

a, b, c, d Indicate significant differences (P<0.05) between: ^a^Type 1, ^b^Type 2, ^c^Type 3, and ^d^Type 4.

In the standing position, thoracic kyphosis angles were significantly greater in types 2 (58.64°) and 3 (58.03°), and smaller in type 1 (39.24°) compared to those in types 4 (51.55°) and 5 (52.32°). In addition, lumbar lordosis and Sac/Hip angles were significantly greater in type 3 (−31.61° and 13.96°, respectively) compared with those in types 1 (−22.58° and 11.32°) and 2 (−21.49° and 8.16°), with type 4 (13.95° and −2.35°) and type 5 (−8.61° and 4.08°) being the smallest ones. Finally, the angle of the whole trunk was greater in types 4 (22.50°) and 5 (10.20°) compared with type 2 (6.10°), with types 1 (1.82°) and 3 (2.99°) being the smallest. Spine inclination, defined by the angle of the whole trunk, showed types 1 and 3 to have the worst balance followed by types 2, 4 and 5. Data is summarized in Table 1 as mean and standard deviation values.

**Figure 2:**
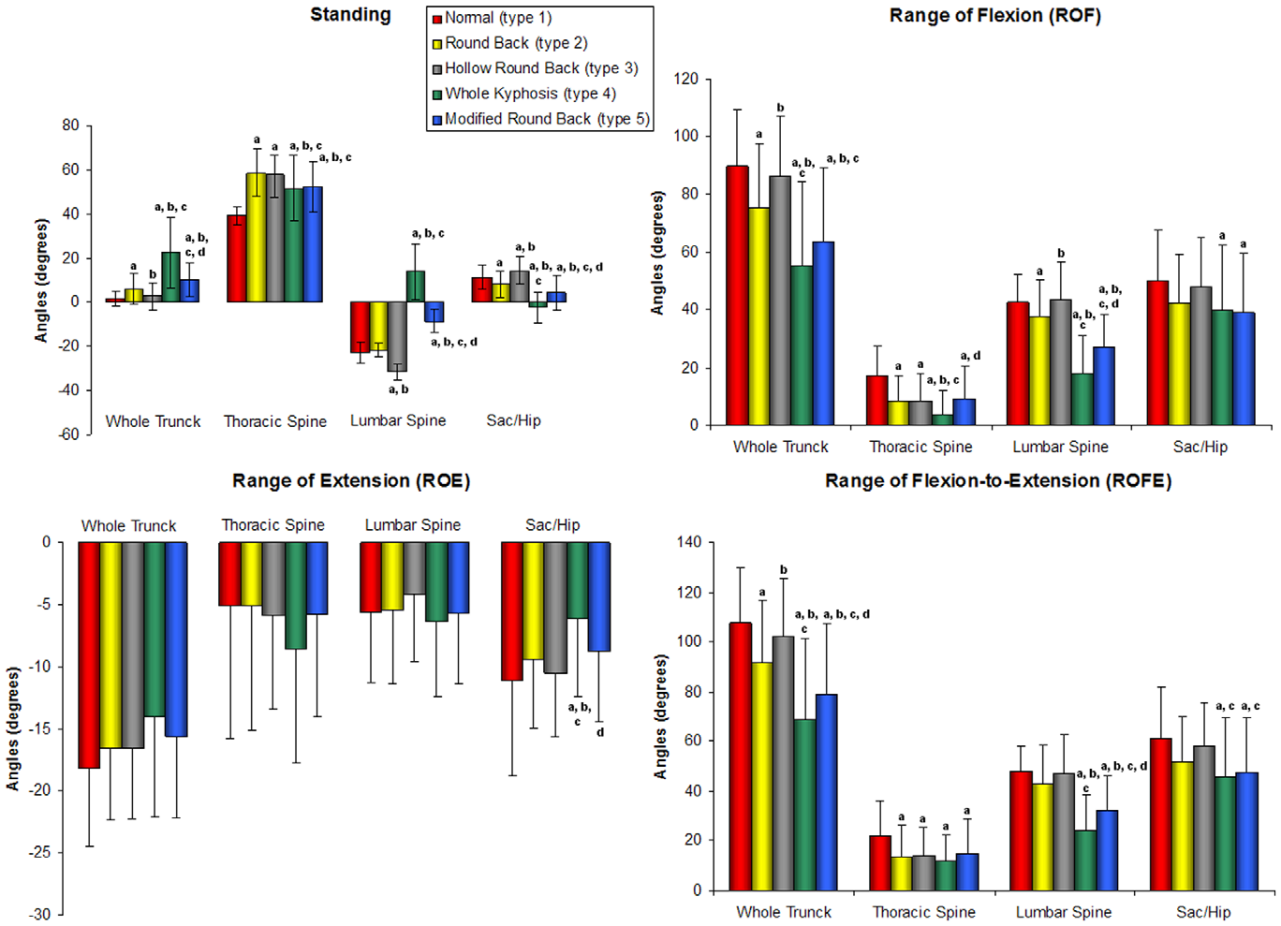
Angle data at different spine sections during standing, flexion and extension conditions. Standing: stand angle in standing; ROE: range of extension; ROF: range of flexion; ROFE: range of flexion-extension. ^a, b, c, d^ Indicate significant differences (P<0.05) between: ^a^Type 1 (n = 50), ^b^Type 2 (n = 173), ^c^Type 3 (n = 75), and ^d^Type 4 (n = 20). Modified type 5 (n = 132).

The range of flexion (ROF) and range of flexion-extension (ROFE) of types 2 and 3 were usually greater than types 4 and 5, with type 1 being the largest. The range of extension (ROE) showed almost no difference in all posture types for the whole spine and individual segments, except on the Sac/Hip angles. These results are described in Table 1 and pictured in Figure 2.

## Discussion

Abnormal posture and spinal mobility of the sagittal plane has been demonstrated to cause significant impairments in the elderly [3], [11], [15]. Prior studies have proven an existing, although conflicting, evidence linking different spinal postures to low back pain [27], [32]. Recently, spinal posture and mobility have been established as important factors linked to quality of life (QOL) in the osteoporotic population [3], [6], [15]. Notably, most of the literature studies focus on either thoracic kyphosis or lumbar lordosis, but not on the change of the entire spinal alignment [3], [11], [13], [15]. Also, there are still some controversies regarding whether the curvature and mobility of the lumbar region better relate to spinal function and balance compared to the thoracic spine, in both cases without fully understanding their progression [6], [8], [9], [13], [17], [26]. Thus, it is meaningful to focus more attention on the global change of the spine and the relationship between the different spinal postural types.

Due to the large degree of variability in sagittal spinal alignment and relatively little work performed toward a classification of osteoporosis in sagittal spinal alignment, the comprehensive classification system and criteria are still ambiguous and equivocal. Roussouly et al. classified patients into four types mainly according to the reciprocal relationships between the sacral slope and the characteristics of the lumbar curvature [33]. Similarly, Lee et al. grouped 86 volunteers into three types based on the horizontal lumbar level [34]. Smith et al. established four subgroups by cluster analysis of three angular measurements of thoraco-lumbo-pelvic alignment [35]. Although those classifications are based on the overall sagittal pattern, the subjects are adolescent or middle-aged patients with or without low back pain who present different geometrical and physiological characteristics compared to an osteoporotic population. In the year 1889, Staffel arranged senile posture into five types: normal, round back, flat back, lordotic back and kypholordotic back, a classification still used at present [28]. Later, Wiles proposed five categories of the human posture based on a combination of the pelvic inclination and dorsolumbar kyphosis [36]. One of these types, round back, was then divided into two additional types according to the lower lumbar curve. Takemitsu et al. classified 105 patients into five types to study the relationship between posture and low back pain [32]. However, due to the complexity of the classification, they were barely used in mass examination studies. Furthermore, a classification system defined by Satoh et al. grouped 73 postmenopausal osteoporotic patients into five groups according to changes of the physiological thoracic and lumbar curvature [27]. Satoh's classification system was used in our study and proved to cover the whole range of our postmenopausal osteoporotic population.

In spite of the percentages of spinal types in our study differing from other literature results, there also exist substantial differences among previous published literature. In this study, type 2 (38.44%) was the most common spinal deformity, compared to the postural type 3 in Satoh’s and Itoi’s studies, 35.6% and 26% respectively [27], [37]. Miyakoshi’s study also presented a higher percentage (26.11%) of type 2 [15]. Moreover, Hongo et. al. suggested differences between a population from Minnesota, USA and a group from Japan, with the former presenting a typical type 2 (hollow round back) and the latter a single kyphotic or lower kyphosis apex [29]. However, until now, research has been done on small population cohorts, making it difficult to obtain decisive relations underlying postural deformity. For this reason, more studies from different ethnic groups, environments and populations are needed.

Most importantly, in addition to not finding Satoh’s type 5 classification on our population, we found a new spine alignment. Having the second highest ratio of spinal deformity (29.33%) and consisting of an increased thoracic kyphosis and decreased lumbar lordosis, we classified this new spine posture as the *modified type 5* or *Modified Round Back.* Reasons for the differences among populations of different geographic areas are multiple, but some of this variability may be related to lifestyle and genetic background.

There exists substantial disparity in the literature regarding the curvature of the thoracic and lumbar spines. Thoracic kyphosis has been reported to be in the 30–50 degrees range, while lumbar lordosis ranges from 20 to 60 degrees [3]–[7], [9]–[11], [13], [15]–[19]. Comparatively, our study reports mean values of 54.21 and 17.95 degrees, respectively. Thus, compared to other geographical places, Chinese women seem to show more thoracic kyphosis with less lumbar lordosis, although many other reasons such as measurement technique, percentage of sex distribution and physical and anthropometric condition could also contribute to this difference.

Spinal deformity has a significant impact on balance disorder and fall in the osteoporotic population [6], [8], [9], [13], [17], [26]. The angle of the whole trunk in our study showed type 3, with a large thoracic kyphosis, to have the best balance, in contrast to type 4, presenting a mild thoracic kyphosis, and type 2, with a large thoracic kyphosis but normal lumbar lordosis, having the worst balance. This is due to the fact that the increased thoracic kyphosis (type 2- Round Back) is readily compensated by increasing lumbar lordosis, resulting in the formation of the type 3 (Hollow Round Back). If progressing round back cannot be compensated by either a reduced lumbar lordosis (type 5) or a kyphotic lumbar spine (type 4), then the spinal balance decreases progressively from worse (type 5) to worst (type 4). These results suggest that it is meaningful to focus more attention on the global change and relationship between different spinal types and balance, rather than “local” changes either thoracic or lumbar.

In this study we provided not only the mobility of individual regions of the spine but also of the whole trunk in both flexion and extension. In addition, the total mobility, from flexion to extension was also shown for individual regions and the whole spine. Our study showed an average range-of-flexion-to-extension (ROFE) of 90.63 degrees for all types, compared to previous studies which show a range of 68–116 degrees [6], [8], [13], [15], [16], [31]. Our findings substantiate prior research showing that spinal mobility decreased in the elderly with postural deformities compared to normal (control) postures. However, we found that the change of spinal mobility was not directly accompanied with a change in thoracic or lumbar curvature, as previous studies have described [6], [8], [9], [13]. For instance, type 3, with nearly most thoracic kyphosis and lumbar lordosis has the same mobility as type 1 without significant differences. On the other hand, as previously stated, those groups without compensation in curvature, either thoracic or lumbar, will have worse mobility. Because spinal mobility is best correlated with quality of life and function, prevention and therapy should be applied, especially for types 4 and 5 in Chinese elderly persons.

Spine curvature and balance is also affected by pelvic orientation and position in the sagittal plane [38]–[40]. When a spine deformity with sagittal imbalance occurs, compensatory mechanisms include not only the spinal column but also the pelvis expressed by sacral slope (SS), indicating the position of the pelvis in the sagittal plane. Moreover, it is much easier to use the SS as an isolated parameter of pelvic orientation, since the measurement of the SS does not require the femoral heads to be visible on standing films [41]. It is commonly reported as a compensatory mechanism: “when the spine tilts forward due to age-related changes, sagittal imbalance, loss of lordosis or increase of kyphosis, the subject will try his/her best to maintain a minimum amount of energy posture and to keep the spine as vertical as possible” [40], [41]. One way to maintain this spino-pelvic alignment is to retrovert the pelvis (decrease of SS) that may be seen as a backward rotation of the pelvis around the hips. In addition, correlations between the various parameters of lumbar and pelvic alignment indicate the sacral slope to be most associated with lumbar lordosis [42]–[44]. Our results are consistent with past observations, as the sacral slope decreases in types 4 and 5, accompanied with a reduction of lumbar lordosis. Also, in type 2, although there is no change in lumbar lordosis, as thoracic kyphosis increased, the sacral slope decreased in order to maintain sagittal balance. Type 3 is the only type with an increased lumbar lordosis and sacral slope. Sacral slope has been reported in only a few other studies using skin-surface devices, and the values obtained in the present study using the spinal mouse compare favorably with past research (all approx.. −13∼23°) [45], [46]. However, these values are smaller than those measured in X-ray films (22∼56°) [39]–[44]. The main reason for this difference may be subject recruitment, as our study involved osteoporotic elderly women, while their research population consisted on young or asymptomatic adults. Furthermore, Barón had shown western population to have a significant lager SS than Asian population [47]. Therefore, in addition to instrumentation use, ethnicity also plays an important role in the differentiation of anthropometric values.

This study presents several limitations. First, there might have been some overlap between spinal types as spinal postural classification was based on changes in thoracic and lumbar curvatures, and there exist a wide range of curvatures. For this reason, the different curvatures types based on the angle change should be clearly and precisely defined to be useful. Second, since this is a cross-sectional study, we were not able to establish any cause-effect relationships and we are not able to verify the time point where the change in curvature occurred. Future longitudinal studies looking at different time point sequences should be undertaken to answer this question. Third, other factors such as body mass index, secondary effects of other fracture types (i.e. wrist and ribs), and exercise level were not recorded thus preventing their analysis on the effect of spinal postural deformities. Finally, position and anatomic pelvic parameters include not only the sacral slope (SS), but also pelvic tilt and pelvic incidence, which are proven to be associated with changes in pelvic spatial orientation and position [38], [48]. However, our results only show SS as this was the only parameter that we could assess with the spinal mouse. All of these factors, as well as knee flexion during gait analysis, should be considered in future studies to confirm their influence in postural deformities, spinal mobility and QOL.

In conclusion, for the first time, the present study classified and compared the mobility and curvature in a Chinese population based on the entire spinal alignment. Types 4 and 5 were shown to have the worst balance in the Chinese elderly population, while type 3 demonstrated the best balance and mobility. We believe that future studies should look into the global spine change when trying to understand postural deformity and function. We also believe that by doing so, it may serve as a convenient clinical marker signaling the falling risk and need for treatment strategies, including exercise and bracing which have shown to be useful for improving balance. Because spinal mobility was best correlated with quality of life and function, prevention and therapy should be especially applied to types 4 and 5 in the Chinese elderly population.
